# Non-Ketotic Hyperglycemia Causing a Transient Unilateral Homonymous Hemianopia: A Manifestation of Occipital Lobe Seizure

**DOI:** 10.7759/cureus.8527

**Published:** 2020-06-09

**Authors:** Salem Gaballa, Kyaw M Hlaing, Safa Moursy, Ameenjamal Ahmed, Avan AlJaf

**Affiliations:** 1 Internal Medicine, LewisGale Medical Center, Salem, USA; 2 Internal Medicine, Lewisgale Medical Center, Salem, USA

**Keywords:** occipital lobe seizure, unilateral homonymous hemianopia, normal brain imaging, normal eeg, nonketotic hyperglycemia, type 2 diabetes

## Abstract

Focal seizures related to non-ketotic hyperglycemia (NKH) are rare in clinical practice. Plasma glucose levels are usually above 16.6 mmol/L and with normal or slightly elevated serum osmolality. The occurrence of focal seizures may be augmented by the absence of ketoacidosis. Electroencephalogram (EEG) during seizures usually confirms the diagnosis, however, the absence of epileptiform discharges does not rule out seizures. A non-ketotic hyperglycemia-associated occipital lobe seizure can manifest itself as color flashes, blurry vision with periodic confusion, and usually resolves with insulin treatment and rehydration. We are reporting a 65-year-old male patient who presented with intermittent confusion and left-sided visual disturbances, found to have a blood glucose of 33.7 mmol/L with a normal anion gap of 10 and calculated serum osmolality of 303 mOsm/L. The patient's visual disturbances responded very well to rehydration and insulin treatment.

## Introduction

Non-ketotic hyperglycemia (NKH)-related epileptic seizures can be diagnosed when high blood glucose is accompanied by normal plasma osmolality and negative urine ketone [1]. The disease pathogenesis is not entirely clear. The possible mechanisms are hyperglycemia or hyperosmolality, a low level of gamma amino-butyric acid (GABA), and focal ischemia. Occipital lobe seizures have been described in the form of colored flashes or hallucinations, sometimes associated with aversive phenomena of the eyes and head.

## Case presentation

A 65-year-old African American patient with a past medical history of anxiety disorder, chronic back pain, hypertension, and untreated Type 2 diabetes mellitus was brought in to the emergency department by his wife, complaining of intermittent confusion and episodic left visual disturbances for a week. The patient also reported a sharp headache around the left periorbital area, rating 5 out of 10 in severity. He also reported seeing blue/red rings occasionally associated with an unsteady gait. He endorses polyuria, polydipsia, polyphagia, and 20 lbs weight gain. He denied any focal weakness or sensation changes, neck stiffness, photophobia, and phonophobia. He denied a smoking history, recreational drug use, or any family history of seizure or stroke. On physical examination, the patient appeared confused, with a slow verbal response to questions. Dense left temporal visual field loss, with an unsteady gait, episodic speech arrest, inattentiveness, and poor immediate memory, was also present on physical exam. There was no focal weakness or deficit, no facial asymmetry, and no sensory deficits.

Complete blood count was within normal limits. The basic metabolic profile was within normal limits except that the blood glucose level was 33.7 mmol/L (normal range 4-5.4 mmol/L), Na 132 mEq/L (corrected Na 140 mEq/L ), carbon dioxide 20.1 mmol/L (22-28 mmol/L), and a normal anion gap of 10. The liver profile was within normal limits. Calculated serum osmolality was 303 mOsm/L (normal range 285-295 mOsm/L), glycated hemoglobin (HbA1C) was found to be 11.5 %. Erythrocyte sedimentation rate was <10 mm/h (his age-adjusted normal range <20 mm/h), C-reactive protein (CRP) was 3.5 mg/L (normal range <3 mg/L), ammonia <10 µ/dL (normal range 15 to 45 µ/dL), and thyroid-stimulating hormone (TSH) was 0.6 mU/L ( normal range 0.4-4 mU/L). Arterial blood gas was unremarkable. Urinalysis was unremarkable except for extensive urine glucose without urine ketones. Computed tomography (CT) of the head without contrast was negative for acute bleed and mass effect. CT of the cervical spine showed severe spondylosis of C5-C6, without fracture and subluxation. Chest X-ray showed a normal cardiac silhouette and normal-appearing lungs without infiltration, edema, or effusion. The patient was started on aspirin 81 mg orally and given 10 units of intravenous (IV) regular insulin and was started on an insulin drip at 250 ml/hour with ½ normal saline. He was later transitioned to 15 units of basal glargine and a low dose of sliding scale insulin. Blood glucose reduced to 200-300 over the next few hours, with almost complete resolution of his visual disturbance and confusion.

Neurology was consulted and recommended a brain magnetic resonance imaging (MRI), which showed no acute stroke but chronic small vessel ischemic changes. Magnetic resonance angiography (MRA) of the head and neck showed no aneurysm or large branch occlusion and widely patent neck vasculature. Electroencephalogram (EEG) showed no epileptiform activity. The patient was started on a moderate-intensity statin and lisinopril 5 mg daily. Endocrinology was consulted and they recommended increasing the basal glargine to 35 units at bedtime and to start metformin 500 mg bid. The patient continued to complain of episodic visual loss with confusion while blood glucose was in the 250-350 range; however, after tighter control of blood glucose with a target of 120-140, his symptoms seemed to improve. Levetiracetam was started upon the neurology's recommendation, with a loading dose of 2000 mg followed by 500 mg bid. The patient's visual disturbances significantly improved and became less frequent.

The ophthalmologist was consulted and noted a normal fundus and retinal examination. Both the neurologist and ophthalmologist agreed on possible occipital lobe seizures due to non-ketotic hyperglycemia. Due to the unavailability of a continuous 24 hours of video EEG monitoring, no epileptic activity was recorded. The patient was discharged 10 days later and was seizure-free on his three-month follow-up. Therefore, levetiracetam was discontinued.

## Discussion

The onset of epileptic seizures in patients aged 50 years or more suggests a brain lesion as the initial hypothesis. One of the possible causes of seizures is a metabolic disorder such as nonketotic hyperglycemia in type 2 diabetes. Focal seizures induced by hyperglycemia were first reported in 1965 [1]. Nonketotic hyperglycemia can vary from asymptomatic to severely symptomatic, and its rapid recognition is vital, as treatment with insulin and aggressive rehydration can prevent serious outcomes [2-3]. Diagnosis is also essential for the management of seizures because they are usually refractory to antiepileptic agents. In fact, some seizure medications, such as phenytoin, may even aggravate them. These seizures typically stop spontaneously after hyperglycemia is corrected [4]. Occipital lobe seizures have been described in the form of colored flashes or, more rarely, elaborate hallucinations sometimes associated with aversive phenomena of the eyes and head [5]. In addition, there are reports of aphasia associated with partial motor effects, pilomotor, and gyratory seizures [6-7].

Seizures associated with nonketotic hyperglycemia are often recurrent, and states of “petit mal” are seen in the form of epilepsia partial continua (EPC) [8-9]. In these cases, the seizures tend to occur at an early stage of hyperglycemia while osmolality is still normal or only slightly elevated. Seizures usually stop once hyperglycemia is under control.

The pathophysiology of epileptic seizures during nonketotic hyperglycemia remains unclear. The possible mechanisms are hyperglycemia or hyperosmolarity, a low level of gamma amino-butyric acid (GABA), and focal ischemia. Brick et al. suggested that the Krebs cycle in NKH is inhibited, resulting in increased GABA metabolism, which decreases GABA levels, thus lowering the seizure threshold [10]. Another hypothesis involved the decrease of seizure threshold due to metabolic disturbances [11]. Hyperosmolality and dehydration induced by hyperglycemia or hypo-sodium accompanying hyperglycemia were suggested to trigger focal seizures and lead to a neurological deficit in some patients [12]. Hyperglycemia creates a hyperosmolar gradient between the intra and extracellular neuronal environments, thereby facilitating seizures. In addition to that, hyperglycemia increases GABA metabolism and thereby diminishes the seizure threshold [12-14]. A few case reports hypothesize that hyperglycemia may cause transient focal ischemia and may thus explain the postictal deficit observed and even reveal or provoke the epileptogenicity of a pre-existing cerebral lesion. Given the rarity of the associated vascular injuries, the latter hypothesis does not appear very convincing [15].

The role of hyperosmolality is unclear. In animal experiments, it can induce seizures in the presence of focal cortical injuries [16]. Nonetheless, imagery is usually normal, and osmolality is not always elevated. The absence of ketosis is also essential; seizures in patients with ketoacidosis are very rare. The acidosis increases the bioavailability of GABA by increasing the activity of the enzyme that syntheses it and by decreasing its transamination [17]. GABA bioavailability is reduced in the absence of ketoacidosis and, therefore, the seizure threshold is minimized. This role of acidosis is useful in the treatment of some pediatric partial seizures, and these patients are prescribed a ketogenic diet [18].

**Figure 1:**
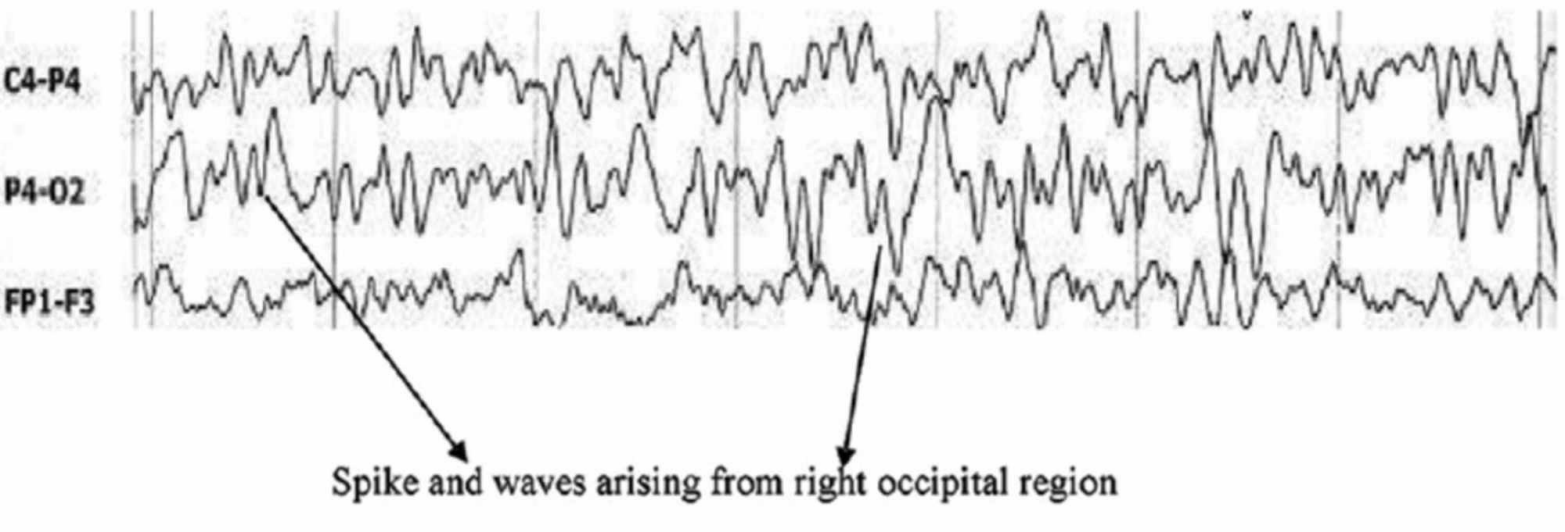
EEG showing spike and waves arising from the right occipital region, suggestive of occipital lobe seizures Courtesy: Dr. Muhammed Jasim Abdul Jalal EEG: electroencephalogram

**Figure 2:**
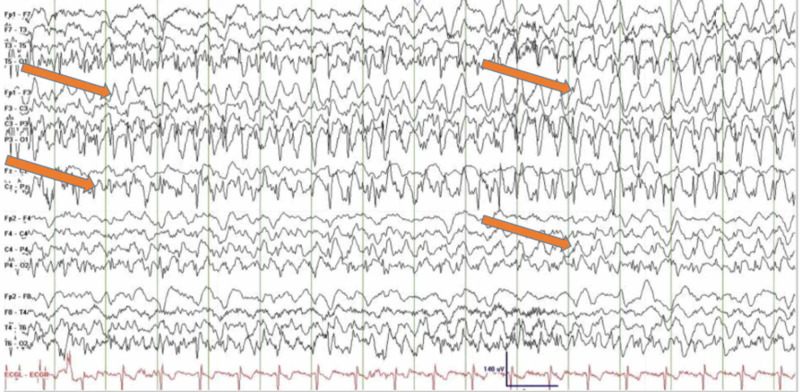
EEG recordings showing the left occipital polyspikes with spread to the right occipital region followed by diffuse bilateral involvement Courtesy: Dr. Swapna L, et al. EEG: electroencephalogram

**Figure 3:**
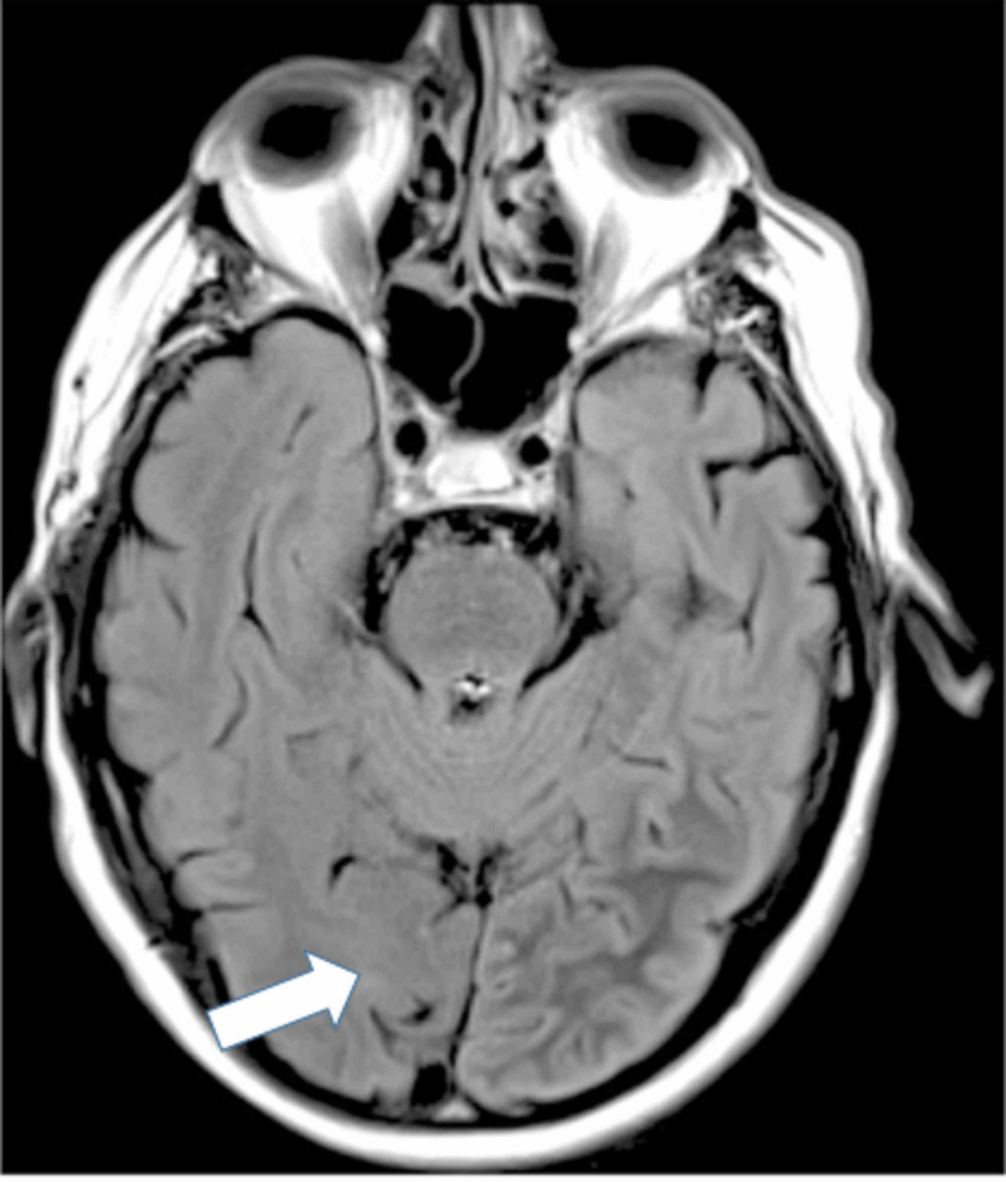
MRI of the brain demonstrating left parieto-occipital subcortical T2 hypointensity on fluid-attenuated inversion recovery in a patient with NKH-related occipital lobe seizure Courtesy: Dr. Swapna L, et al. MRI: magnetic resonance imaging; NKH: non-ketotic hyperglycemia

**Figure 4:**
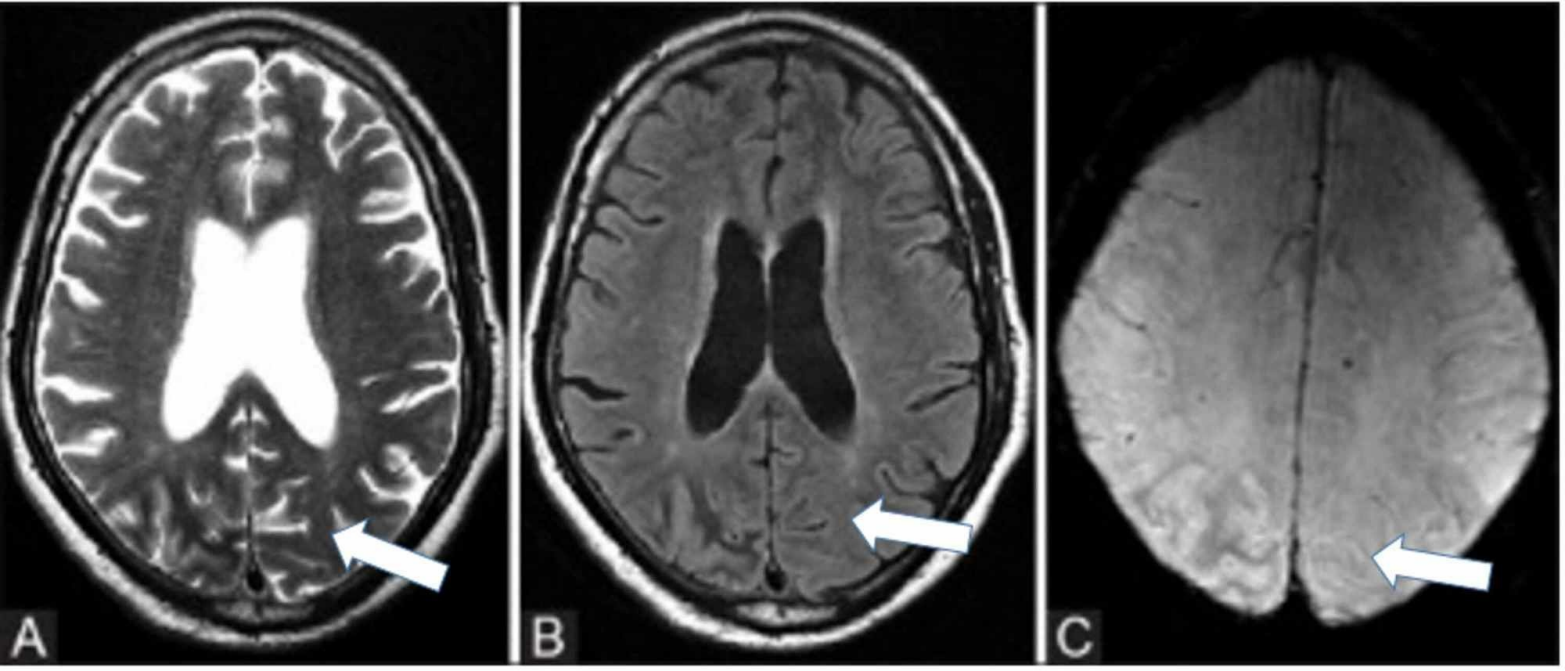
Axial T2W (A), FLAIR (B), and SWAN (C) images of the brain show cortical hyper-intensity on T2W, FLAIR, and SWAN images involving the right parieto-occipital region with adjacent subcortical white matter hypo-intensity in a patient with NKH-related occipital lobe seizure Courtesy: Dr. Shivaprakash B Hiremath T2W: T2 weighted; FLAIR: fluid-attenuated inversion recovery; SWAN: star-weighted angiography radiology; NKH: non-ketotic hyperglycemia

EEG findings during occipital lobe seizures usually confirm the diagnosis, as shown in Figures 1-2; however, the absence of periodic epileptiform discharges has been reported in the literature. Brain imaging is usually unremarkable, but subcortical T2 hypointensity has been reported, as shown in Figures 3-4.

Using antiepileptic medications is generally not justified and sometimes is likely to aggravate seizures. Phenytoin, in particular, should not be used, as it inhibits insulin secretion. Moreover, cases of EPC are typically unresponsive to antiepileptic medications. Meanwhile, the rapid correction of hyperglycemia stops seizures very effectively [19].

## Conclusions

Seizures related to NKH are usually partial seizures. Transient homonymous hemianopia is not an uncommon manifestation of an occipital lobe seizure, often with normal neuroimaging and EEG. The visual field deficits associated with NKH are usually reversible with rehydration and insulin treatment, with or without antiepileptic medications.
